# Structural Stereochemistry of Androstene Hormones Determines Interactions with Human Androgen, Estrogen, and Glucocorticoid Receptors

**DOI:** 10.1155/2013/203606

**Published:** 2013-04-04

**Authors:** Thomas L. Shaak, Dayanjan S. Wijesinghe, Charles E. Chalfant, Robert F. Diegelmann, Kevin R. Ward, Roger M. Loria

**Affiliations:** ^1^Department of Integrative Life Sciences, Virginia Commonwealth University, Richmond, VA 23298, USA; ^2^Virginia Commonwealth University Reanimation Engineering Science Center (VCURES), Virginia Commonwealth University, Richmond, VA 23298, USA; ^3^Department of Biochemistry, Virginia Commonwealth University, 1101 E. Marshall Street, Sanger Hall, Room 2-004, Richmond, VA 23298, USA; ^4^Hunter Holmes McGuire VA Medical Center, Richmond, VA 23249, USA; ^5^Department of Biochemistry and Molecular Biology, Virginia Commonwealth University, Richmond, VA 23298, USA; ^6^Massey Cancer Center, Richmond, VA 23298, USA; ^7^Michigan Critical Injury and Illness Research Center, Department of Emergency Medicine, University of Michigan, Ann Arbor, MI 48109, USA; ^8^Department of Microbiology, Immunology, Pathology and Emergency Medicine, Virginia Commonwealth University, Richmond, VA 23298, USA

## Abstract

DHEA, 17*α*-AED, 17*β*-AED, and 17*β*-AET exhibit strong biological activity that has been attributed to androgenic, estrogenic, or antiglucocorticoid activity in vivo and in vitro. This study compared DHEA, 17*α*-AED, 17*β*-AED, and 17*β*-AET for their ability to activate the human AR, ER, and GR and determine the relative androgenicity, estrogenicity, and glucocorticoid activity. The results show that, at the receptor level, these androstene hormones are weak AR and even weaker ER activators. Direct androstene hormone activation of the human AR, ER*α*, and ER*β* may not be essential for their biological function. Similarly, these hormones indirectly activated the human GR, only in the presence of high dexamethasone concentrations. These results underscore the major difference between androstene hormone interactions with these nuclear receptors and their biological effects.

## 1. Introduction

DHEA, an androstene hormone, has been shown to possess a wide range of beneficial biological effects mainly attributed to immune system modulation [1]. DHEA is metabolized into more active metabolites, that is, 17*β*-AED and 17*β*-AET, as well as testosterone and estradiol [1, 2]. 17*β*-AED and 17*β*-AET have been reported to prevent the morbidity and mortality of otherwise lethal infections [3, 4], potentiate lymphocyte activation, and counteract the immune suppressive action of hydrocortisone [5–7], thus leading to beneficial effects in diverse human diseases including resistance to infection, neuroprotection, wound healing, diabetes, hepatic injury, cardiovascular disease, and cancer [8–10].

17*α*-AED mediates autophagy of glial and breast cancers and apoptosis of myeloid tumor cells [11–13]. 17*β*-AED and 17*α*-AED naturally exist in epimeric forms based on whether the hydroxyl group is above (*β*) or below (*α*) the Δ^5^ cycloperhydrophenanthrene ring. Addition of a hydroxyl group at the C7 position to 17*β*-AED results in the formation of Δ^5^-androstene-3*β*, 7*β*, 17*β*-triol (17*β*-AET). The biological activities of 17*α*-AED, 17*β*-AED, and 17*β*-AET have exhibited a structure-activity relationship that depends on the orientation and location of the hydroxyl groups [13].

Androstene hormones (AH) have been shown to promulgate their biological effects in many different animal models including mice, rats, monkeys, and some specific human tissues. Reports have associated the mechanism of action of androstene hormone metabolites with androgen, estrogen, and glucocorticoid receptor activity [14–16]. Adrenal hormones have been shown to activate both androgen and estrogen constructs. In this regard, it has been documented that 17*β*-AED can activate the AR in prostate tissue in the presence of commonly used antiandrogens [17]. Inhibitors of both the androgen receptor and the estrogen receptors demonstrated that AR and ER*β* receptors combine to affect gene transcription [18]. Additionally, 17*β*-AED was recently shown to be a part of an anti-inflammatory mechanism that utilizes the ER*β* [19]. 17*β*-AED and 17*β*-AET have been documented in vitro and in vivo to oppose the action of hydrocortisone indicating that there may be crosstalk with the GR [6, 20, 21].

DHEA has been shown to possess weak androgenicity and estrogenicity [22]. Because 17*α*-AED, 17*β*-AED, and 17*β*-AET are more potent metabolites of DHEA that exhibit strong biological activity that could be attributed to androgenic, estrogenic, or antiglucocorticoid activity in vivo and in vitro, it was advantageous to identify whether or not this is directly mediated by the human ER, AR, and GR at the cellular level. Additionally, androstenediol has been modeled as a chemical with a 3*β*-hydroxy and a saturated A ring which can act as an estrogen [23]. Consequently, we compared DHEA, 17*α*-AED, 17*β*-AED, and 17*β*-AET in the Indigo Biosciences nuclear receptor assay system for their ability to activate the human AR, ER, and GR and determine the relative androgenicity and estrogenicity of these androstene hormone derivatives.

## 2. Materials and Methods

### 2.1. Nuclear Receptor Transactivation Assays

Nuclear receptor transactivation assays were obtained from Indigo Biosciences (State College, PA, USA) and were utilized to assess the activation of human AR, ER*β*, and ER*α*. receptors. Briefly, stocks of the compounds tested were prepared and diluted in a medium provided by the manufacturer. Cell medium was tested for hormone activity by mass spectrometry (Section 2.3). Frozen reporter cells provided in the assay kit were thawed and compound dilutions were added immediately. Cells were incubated for 24 hours and the activation response was measured on a luminometer (Perkin-Elmer, MA, USA). The cells consisted of non-human mammalian cells engineered by Indigo Biosciences to provide constitutive high-level expression of full length, unmodified human androgen receptor (NR3C4), human estrogen receptor 1 (NR3A1), human estrogen receptor 2 (NR3A2), and full length, human glucocorticoid receptor (NR3C1).

The nonhuman mammalian reporter cells included a luciferase reporter gene functionally linked to a human nuclear receptor-responsive promoter. The cells are engineered so that only interactions with the human receptor will induce luciferase expression in the treated reporter cells to quantitate nuclear receptor activation. Positive control ligand performance was measured by the manufacturer and provided in the technical manuals thus allowing accurate comparison for assay performance. Additionally, the control ligands of the receptors (testosterone, 17*β*-estradiol, and dexamethasone) were tested on the same test plates (*n* = 3 to allow statistical analysis) with the androstene hormones and controls.

### 2.2. Preparation of Stock Hormone Solutions

Stock solutions of 17*β*-AED, 17*α*-AED, and testosterone (Sigma-Aldrich, St. Louis, MO, USA) were prepared by dissolving the compounds into 100% ethanol to a final 50 mM stock solution concentration; 17*β*-AET was also a stock solution of 50 mM but was dissolved into 1 : 1 DMSO/ethanol because of solubility issues. Cyproterone acetate (Sigma-Aldrich) was diluted with 100% ethanol and used at a 10 uM concentration. All stock solutions were diluted to final concentrations using the dilution fluid provided in each kit. All tests were performed with negative controls on the same plate and contained media alone and media containing the same amount of ethanol utilized in the stock solutions. All assay control results were in accordance with the stated technical performance specifications.

### 2.3. Mass Spectrometry

LC-MS/MS analyses of the steroid hormones were carried out using a Shimadzu Nexera UPLC device coupled (Shimadzu Corp, Kyoto, Japan) to AB Sciex (Foster City, CA, USA) 5500 Hybrid Triple Quadrupole Linear Ion Trap Mass Spectrometer operating in multiple reaction monitoring mode. Nitrogen produced by a high-purity nitrogen generator (PEAK Scientific Instruments Ltd, Chicago, Il, USA) was used as curtain, nebulizer, and collision gases. Unit mass resolution was set in both mass-resolving quadrupole Q1 and Q3. Ionization of the analytes was carried out using an APCI source. Multiple MRM transitions were selected for each analyte to eliminate ambiguity in analyte identification. For all steroids other than AED, 25 microliters of the media was directly injected onto a 2.1 × 50 mm 2.6 *μ*m C18 reverse phase column (Phenomenex) and was separated via a linear gradient of water : methanol 98 : 2 (Solvent A) to methanol : water 70 : 30 (Solvent B). Both solvents contained 5 mM ammonium formate with 1% formic acid. Separation of 17*α*-AED and 17*β*-AED was carried out using 2.1 × 150 mm 2.6 *μ*m C18 reverse phase column (Phenomenex) and was done using same Solvent A as above and 98 : 2 methanol : water with 5 mM ammonium formate and 1% formic acid as Solvent B. Steroid hormones were detected using precursor product. MRM pairs are as follows: DHEA (271-213, 271-197), androstenetriol (307-158, 307-254), androstenediol (291-95, 291-159, 291-255), testosterone (289-97, 289-109), androstenedione (287-97, 287-109), and 17*β*-estradiol (273-107, 273-135, 273-77). Where there were multiple transitions, the dominant peak was used in the analysis. All analytes demonstrated a minimal limit of detection of at least 0.6 nM.

#### 2.3.1. Cellular Uptake of Androstene Hormones and Normalization of Transactivation Assay Results

It was relevant to determine the relative uptake of each androstene hormone in the Indigo Assay System. Cells and cell medium utilized in the assays were provided by the manufacturer (Indigo Biosciences). The following components were found to be below the limit of detection in the cells and cell medium: DHEA, 17*β*-estradiol, androstenedione, testosterone, 17*α*-AED, 17*β*-AED, and 17*β*-AET. LCMS data was utilized as a ratio between the background subtracted signal (area under the curve of the LCMS trace) at time zero and 24 hours for each analyte investigated. The ratios were then utilized to create normalization factors for the cellular uptake of 17*α*-AED, 17*β*-AED, and 17*β*-AET as compared to DHEA. The normalization factors were DHEA: 1, 17*α*-AED: 1.71, 17*β*-AED: 1.85, and 17*β*-AET: 2.40, respectively. Background activity due to cells, media, and vehicle was subtracted before the data were normalized.

#### 2.3.2. Metabolism of Androstene Hormones

The cell medium was analyzed before and after incubation with assay cells. Mass spectroscopy (Section 2.3) was utilized to detect androstenedione, 17*β*-AED, 17*α*-AED, and 17*β*-AET, as well as testosterone and 17*β*-estradiol that were expressed in the medium. Approximately 9% of DHEA was metabolized to 17*β*-AED after a 24-hour incubation with assay cells. No other DHEA metabolites were detected. Mass spectroscopy did not detect any metabolites of the other androstene hormones in the media after a 24-hour incubation period. The mass spectrometry data show that DHEA was minimally metabolized to 17*β*-AED in this cell construct, but not to testosterone or 17*β*-estradiol (Section 2.3.2). 17*β*-AED, 17*β*-AET, and 17*α*-AED were not metabolized.

### 2.4. Statistical Analysis

All statistical analyses were performed using SigmaPlot version 12 (SSI, San Jose, CA, USA). Hormone EC_50_ level and the estrogen receptor alpha activation statistical analyses were performed with a Student's *t*-test while all other hormone activation statistical comparisons were performed with a one-way ANOVA. *P* value levels < 0.05 were considered significant. Statistics on test groups were done before normalization to cellular uptake and were performed between test groups and controls.

## 3. Results and Discussion

### 3.1. Androstene Hormone Structures

**Figure 1:**
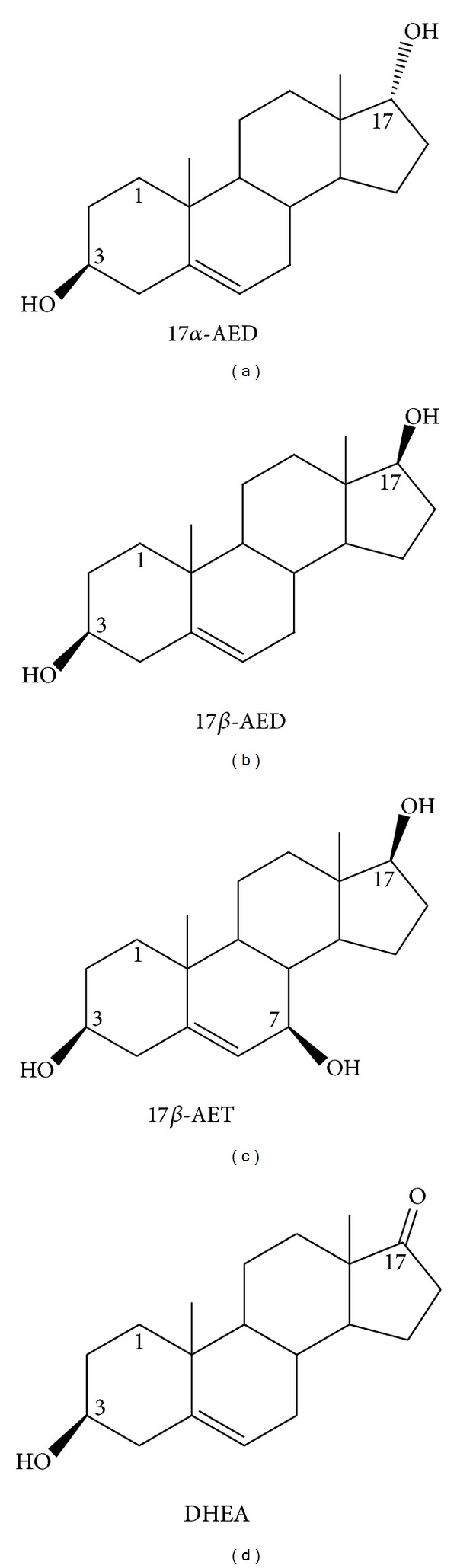
The structures of the androstene hormones. The androstene hormones are shown with the Δ^5^ cycloperhydrophenanthrene ring. All steroids have a C3 hydroxyl group in the (*β*) beta position. The C7 hydroxyl group of androstenetriol is in the *β*-position. The C17 hydroxyl of androstenediol epimers are in either the (*α*) alpha or (*β*) beta position.

The hormones that were used in this study are listed in Figure 1. The structures demonstrate the similarities and unique characteristics of each androstene hormone. The main differences are the orientation of the hydroxyl group at position C17 for 17*α*-AED and 17*β*-AED, the orientation and position of the hydroxyl group at position C7 for 17*β*-AET, and the ketone group at position 17 for DHEA. 17*α*-AED and 17*β*-AED are chemically identical except for the placement of the hydroxyl group in relation to (above or below) the Δ^5^ cycloperhydrophenanthrene ring. All adrenal hormones in this study, with the exception of DHEA, possess hydroxyl groups in the C3 and C17 position with 17*α*-AED having the C17 hydroxyl group in the (*α*) position. This position at C17 results in remarkable biological actions [11, 13] while the hydroxyl group at C3 was shown not to influence the biological activity [24].

### 3.2. Androstene Hormone Activation of the Human AR

**Figure 2:**
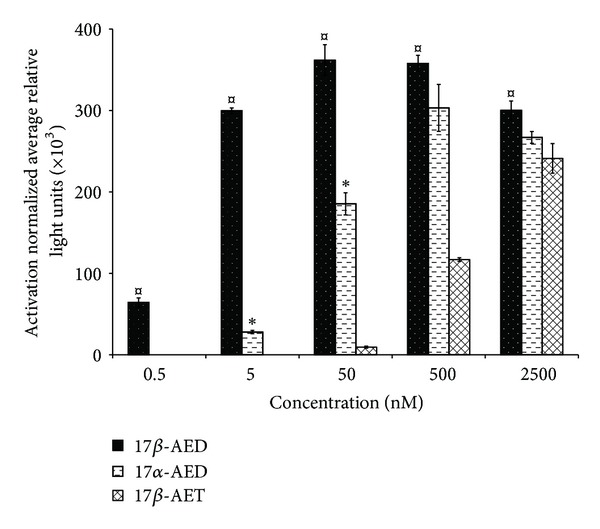
Androstene hormone activation of the human androgen receptor. Reporter cells were treated with androstene hormone metabolites (*n* = 3), incubated for 24 hours, and then assayed for luciferase activity. Androstene hormone activity was normalized to cellular uptake (Section 2.3.1). Error bars, ±1 SD. Statistical significance, *P* < 0.001 from all less activating androstene hormone metabolites (***¤***), *P* < 0.001 from less activating androstene hormone metabolites (∗).

The data demonstrate that both the orientation of the hydroxyl at position C17 and the addition of the hydroxyl at position C7 affected the ability of 17*β*-AED, 17*β*-AET, and 17*α*-AED to activate the human AR construct (Figure 2). The AR construct contains a luciferase reporter gene that is functionally linked to an AR responsive promoter. The luciferase reading is utilized as a surrogate measure for AR binding. The EC_50_ was calculated for testosterone as the 50% activation point. EC_50_ values for the androstene hormones and testosterone were calculated (Table 1) and compared utilizing the ratio of the androstene hormone EC_50_ to testosterone EC_50_ (relative androgenicity). All of the androstene hormones tested showed a significant (*P* < 0.001) reduced androgenicity when compared to testosterone (Table 1). 17*β*-AED had only 1/5th the ability of testosterone to activate the androgen receptor. Changing the orientation of the C17 hydroxyl group on 17*α*-AED resulted in a further reduction to 1/60th the activity as compared to testosterone. Addition of the hydroxyl group to the C7 position further reduced the ability to activate the human AR construct to 1/1326th as that of testosterone. The androstene hormone activation of the human AR was rank ordered based on strength of activation. The rank order was 17*β*-AED ≫17*α*-AED ⋙17*β*-AET (Figure 2). DHEA binding to the AR was excluded from these experiments since its androgenicity has been reported previously [22].

### 3.3. Androstene Hormone Activation of the Human ER*β* and ER*α* Receptors

**Figure 3:**
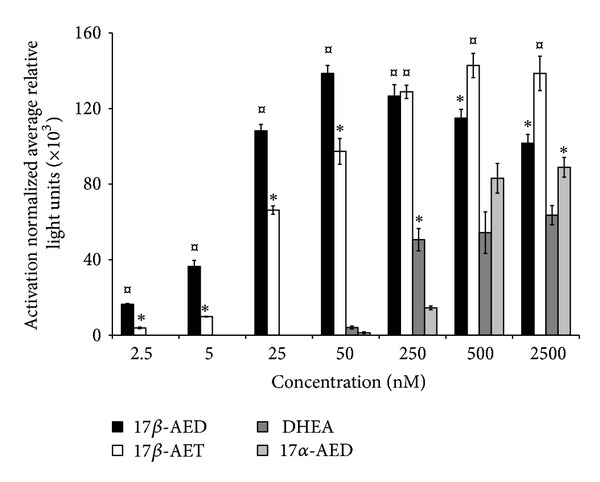
Androstene hormone activation of the human estrogen receptor beta. Reporter cells were treated with androstene hormones (*n* = 3), incubated for 24 hours, and then assayed for luciferase activity. Androstene hormone activity was normalized to cellular uptake (Section 2.3.1). Error bars, ±1 SD. Statistical significance, *P* < 0.001 (***¤***) versus androstene hormone metabolites in the same treatment group, *P* < 0.001 (∗) versus lower reacting androstene hormones in the same treatment group.

The data demonstrate that both the orientation of the hydroxyl at position C17 and the addition of the hydroxyl at position C7 affected the ability of DHEA, 17*β*-AED, 17*β*-AET, and 17*α*-AED to activate the human ER*β* construct (Figure 3). The ER construct contains a luciferase reporter gene that is functionally linked to an ER responsive promoter. The luciferase reading is utilized as a surrogate measure for ER binding. The EC_50_ was calculated for 17*β*-estradiol as the 50% activation point. EC_50_ values for the androstene hormones and 17*β*-estradiol were calculated (Table 2) and compared utilizing the ratio of the androstene hormone EC_50_ to 17*β*-estradiol EC_50_ (relative estrogenicity). All of the tested androstene hormones demonstrated a significantly (*P* < 0.001) decreased estrogenicity compared to 17*β*-estradiol (Table 2). 17*β*-AED had only 1/282nd the ability of 17*β*-estradiol to activate the ER*β*. The orientation change of the hydroxyl group at position C17 of 17*β*-AED to the (*α*) position resulted in 17*α*-AED possessing 1/7609th the ability of 17*β*-estradiol to activate the ER*β* receptor as 17*β*-estradiol. This represents a drastic decrease in estrogenicity from the 17*β*-AED epimer. The presence of the hydroxyl group at C7 of 17*β*-AET resulted in 1/587th the ability to activate the ER*β*. DHEA, with a ketone group in the C17 position, possessed 1/3543rd the ability to activate the ER*β*.

**Figure 4:**
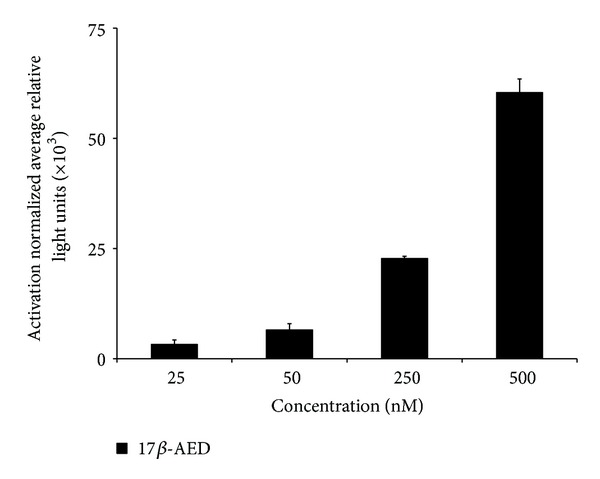
17*β*-AED activation of the human estrogen receptor alpha. Reporter cells were treated with 17*β*-AED (*n* = 3), incubated for 24 hours, and then assayed for luciferase activity. 17*β*-AED activity was normalized to cellular uptake (Section 2.3.1), error bars, ±1 SD.

The rank order of androstene hormone activation on the human ER*β* receptor can be displayed as follows: 17*β*-AED > 17*β*-AET > DHEA > 17*α*-AED (Figure 3). These androstene hormones also specifically activated the human ER*β* receptor and demonstrated the crucial effect of the (*β*) C17 hydroxyl group. 17*β*-AED and 17*α*-AED activated the ER*β* receptor 2 and 3 orders of magnitude lower, respectively, than 17*β*-estradiol (Table 2). The 17*β*-AED demonstrated an estrogenicity of 1/1176 when assayed on the human ER*α*. Activation of the ER*α* receptor by 17*β*-AED did not become apparent until the concentration reached 25 nM (Figure 4) which was 3 orders of magnitude lower than 17*β*-estradiol, further demonstrating the weak estrogenicity displayed by these hormones at the level of the ERs. Finally, it should be noted that the androstene hormones only weakly activated the AR and were even weaker activators of the human ERs.

### 3.4. Androstene Hormones Activation of the Human Glucocorticoid Receptor (GR)

**Figure 5:**
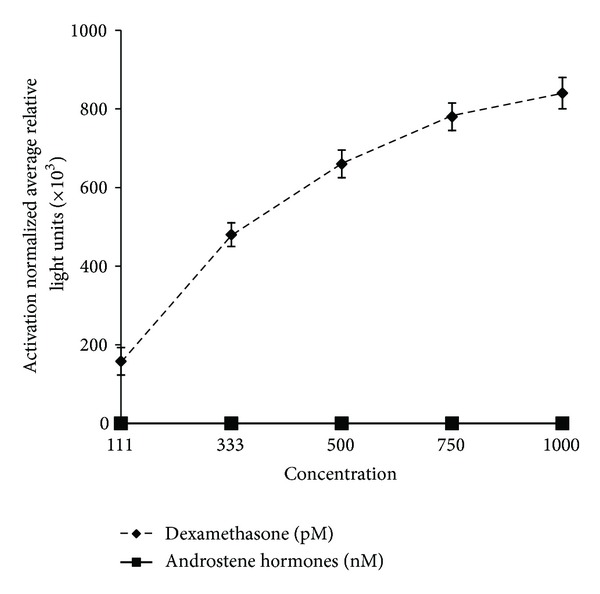
Dexamethasone or androstene hormone activation of the human glucocorticoid receptor. Reporter cells were treated with dexamethasone or androstene hormones alone (*n* = 3), incubated for 24 hours, and then assayed for luciferase activity. Androstene hormones are DHEA, 17*β*-AED, 17*α*-AED, and 17*β*- AET. Error bars, ±1 SD.

**Figure 6:**
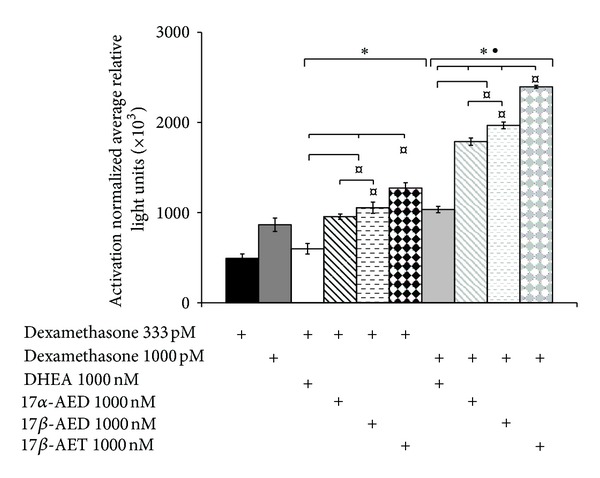
Androstene hormones and dexamethasone activation of the human GR. Reporter cells were treated with dexamethasone alone or a combination of dexamethasone and androstene hormone, incubated for 24 hours, and then assayed for luciferase activity (*n* = 3). Androstene hormone activity was normalized to cellular uptake (Section 2.3.1). Error bars, ±1 SD. Statistical significance, *P* is at least < 0.05 from other androstene hormone metabolites within the dexamethasone concentration treatment group (***¤***), *P* is at least < 0.05 versus control (∗), *P* is at least < 0.05 from 333 pM dexamethasone treatment group (●).

17*β*-AED and especially 17*β*-AET are known to produce significantly affected glucocorticoid activity in vivo [6, 20, 21]. Therefore, the human GR construct response to 17*β*-AED, 17*β*-AET, DHEA, and 17*α*-AED alone and in combination with dexamethasone was evaluated. The results showed that dexamethasone alone activated the human GR while 17*β*-AED, 17*β*-AET, DHEA, and 17*α*-AED alone were negative at all concentrations tested (Figure 5). The human GR was then tested for activity with androstene hormones in the presence of dexamethasone. Unexpectedly, at suprapharmacological levels of dexamethasone there was a considerable activation of the human GR by 1 *μ*M of each of the androstene hormones, which was greater than with dexamethasone alone with a higher activity when dexamethasone concentration was increased from 333 pM to 1000 pM (Figure 6).

The rank order of activation of the dexamethasone-bound human GR in the presence of the androstene hormones is as follows: 17*β*-AET > 17*β*-AED > 17*α*-AED > DHEA. These data demonstrate that the C7 hydroxyl present in 17*β*-AET produced the strongest activation of the dexamethasone-bound human GR. The (*β*) C17 hydroxyl of 17*β*-AED produced a stronger activation than did the (*α*) C17 hydroxyl of 17*α*-AED. Thus, while 17*β*-AED and 17*α*-AED produced unique activation of the dexamethasone-bound human GR, the effect of the C17 hydroxyl group conformation was less apparent. DHEA, which has the keto group in the C17 position, possessed the least ability to activate the dexamethasone-bound human GR.

**Figure 7:**
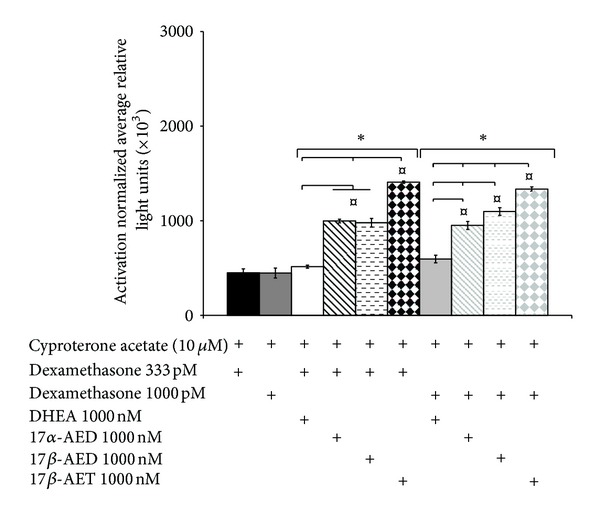
Androstene hormone and dexamethasone activation of the human GR in the presence of cyproterone acetate. Reporter cells were treated with dexamethasone and cyproterone acetate alone or androstene hormones with dexamethasone and cyproterone acetate (*n* = 3), incubated for 24 hours, and then assayed for luciferase activity. Androstene hormone activity was normalized to cellular uptake (Section 2.3.1). Error bars, ±1 SD. Statistical significance, *P* is at least < 0.05 from other androstene hormone metabolites within the treatment group (***¤***), *P* < 0.05 from controls (∗).

We next tested these effects with the dexamethasone inhibitor, cyproterone acetate, to see if we could reduce or eliminate the dexamethasone and androstene hormone activation of the human GR. Cyproterone acetate was selected as the inhibitor because of its unique glucocorticoid receptor inhibiting properties [25]. Cyproterone effectively inhibited the activation of the human GR by dexamethasone (Figure 7). Cyproterone acetate at a concentration of 10 uM was tested in the presence of the androstene hormones alone (1.0 uM) and there was no activation detected (data not shown). Androstene hormones, however, in the presence of cyproterone and dexamethasone exhibited different levels of activation that were significantly increased above the dexamethasone/cyproterone alone controls (Figure 7). These results are of particular clinical significance because it demonstrates that high-dose dexamethasone alters the human GR to interact with other biologically active hormones at the receptor level. Importantly, dexamethasone is known to cause adverse effects in humans [26].

The rank order of activation of the dexamethasone/cyproterone acetate-bound human GR in the presence of these androstene hormones is as follows: 17*β*-AET > 17*β*-AED > 17*α*-AED > DHEA. This rank order of activation on the inhibited human GR was the same as that of the uninhibited human GR indicating that the interaction of the androstene hormones and the dexamethasone/cyproterone-bound human GR was not disrupted. Since cyproterone acetate is a passive inhibitor of the human GR and opposes dexamethasone through an overlapping steroid scaffold mechanism, this suggests that the androstene hormone activation is mediated by an interaction that occurs outside the dexamethasone/cyproterone acetate-bound complex [25]. Additionally, the presence of dexamethasone-bound human GR is required to observe activation by the androstene hormones while cyproterone acetate alone does not mediate this effect. Together these data suggest an indirect activation of the ligand-bound human GR by 17*β*-AET, 17*β*-AED, 17*α*-AED, and DHEA.

## 4. Conclusion

This report indicates that the position of the hydroxyl group at C17 and/or the addition of the hydroxyl group at position C7 significantly affected the ability of 17*β*-AET, 17*β*-AED, 17*α*-AED, and DHEA to interact with the human estrogen, androgen, and ligand-bound glucocorticoid receptors. 17*β*-AET, 17*β*-AED, 17*α*-AED, and DHEA were shown to interact either directly or indirectly with the human AR, ER, and GR. Importantly, 17*β*-AET, 17*β*-AED, 17*α*-AED, and DHEA were shown to possess weak androgenicity and even weaker estrogenicity at the receptor level. Clinically, this is beneficial because the biological effects can be realized without unwanted androgenic or estrogenic effects.

In a stark contrast to the minimal receptor activation of AR, ER, and GR, these same androstene hormones produce striking biological effects in vitro and in vivo which have been attributed to activity with the AR, ER, or GR. Clearly, these effects may not be mediated by the direct androstene hormone interaction with the human AR, ER*α*, ER*β*, and GR. Indeed, the biological mechanism, may not require AR or ER to achieve significant effects [27, 28]. Furthermore, the interaction with dexamethasone is indirect, occurs at high doses, and is not abolished by cyproterone acetate. Taken together, the data shows that interactions of 17*β*-AET, 17*β*-AED, 17*α*-AED, and DHEA with the human AR, ER*α*, ER*β*, and GR are directed by the structure activity of these androstene hormones with minimal androgenic, estrogenic, or glucocorticoid effects and accentuates the need to further uncover the implied yet unidentified main mechanism(s) of action of these important adrenal hormones.

## Figures and Tables

**Table 1:** Relative androgenicity of androstene hormones.

Hormone	EC_50_ (nM)	Androgenicity	*P* value
Testosterone	0.35	1	1
Δ^5^-Androstene-3*β*, 17*β*-diol	1.8	1/5	<0.001
Δ^5^-Androstene-3*β*, 17*α*-diol	21	1/60	<0.001
Δ^5^-Androstene-3*β*, 7*β*, 17*β*-triol	464	1/1326	<0.001

The EC_50_ was calculated as the 50% activation point. The androgenicity is the testosterone EC_50_ divided by the androstene hormone EC_50_. This data does not normalize the cellular uptake of androstene hormones.

**Table 2:** Relative estrogenicity of androstene hormones.

Hormone	EC_50_ (nM)	Estrogenicity	*P* value
17*β*-Estradiol	0.046	1	1
Δ^5^-Androstene-3*β*, 17*β*-diol	13	1/282	<0.001
Δ^5^-Androstene-3*β*, 7*β*, 17*β*-triol	27	1/587	<0.001
DHEA	163	1/3543	<0.001
Δ^5^-Androstene-3*β*, 17*α*-diol	350	1/7609	<0.001

The EC_50_ was calculated as the 50% activation point. The estrogenicity is the 17*β*-estradiol EC_50_ divided by the androstene hormone EC_50_. This data does not normalize the cellular uptake of androstene hormones.

## Abbreviations

17*β*-AED: Δ^5^-androstene-3*β*, 17*β*-diol
17*α*-AED: Δ^5^-androstene-3*β*, 17*α*-diol
17*β*-AET: Δ^5^-androstene-3*β*, 7*β*, 17*β*-triol
Androstene hormones: 17*β*-AED, 17*α*-AED, 17*β*-AET, DHEA
Androstene hormone metabolites: 17*β*-AED, 17*α*-AED, 17*β*-AET
DHEA: Dehydroepiandrosterone
AR: Androgen receptor
ER*α*: Estrogen receptor alpha
ER*β*: Estrogen receptor beta
GR: Glucocorticoid receptor
(*α*): Alpha position
(*β*): Beta position.
